# Native Mass Spectrometry for the Study of PROTAC GNE‐987‐Containing Ternary Complexes

**DOI:** 10.1002/cmdc.202100113

**Published:** 2021-05-04

**Authors:** Louise M. Sternicki, Jim Nonomiya, Miaomiao Liu, Melinda M. Mulvihill, Ronald J. Quinn

**Affiliations:** ^1^ Griffith Institute for Drug Discovery Griffith University 46 Don Young Road Nathan QLD 4111 Australia; ^2^ Genentech Inc. 1 DNA Way South San Francisco CA 94080 USA

**Keywords:** Proteolysis Targeting Chimeras, PROTAC, native MS, GNE-987, high-throughput screening

## Abstract

PROteolysis TArgeting Chimeras (PROTACs) promote the degradation, rather than inhibition, of a drug target as a mechanism for therapeutic treatment. Bifunctional PROTAC molecules allow simultaneous binding of both the target protein and an E3‐Ubiquitin ligase, bringing the two proteins into close spatial proximity to allow ubiquitinylation and degradation of the target protein via the cell's endogenous protein degradation pathway. We utilized native mass spectrometry (MS) to study the ternary complexes promoted by the previously reported PROTAC GNE‐987 between Brd4 bromodomains 1 and 2, and Von Hippel Lindeau E3‐Ubiquitin Ligase. Native MS at high resolution allowed us to measure ternary complex formation as a function of PROTAC concentration to provide a measure of complex affinity and stability, whilst simultaneously measuring other intermediate protein species. Native MS provides a high‐throughput, low sample consumption, direct screening method to measure ternary complexes for PROTAC development.

## Graphical abstract

**MS for PROTACS**! Native mass spectrometry (MS) can measure ternary complexes between PROTACs, target proteins and E3‐ubiqutin ligases, together with other protein species present at equilibrium, with accurate mass. Stable ternary complexes are a requirement for PROTAC‐mediated target protein degradation. Native MS can be used as a high‐throughput screening technique to directly detect PROTAC ternary complexes that will result in efficient target degradation.

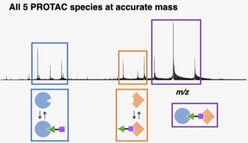

## Introduction

**Figure 1:**
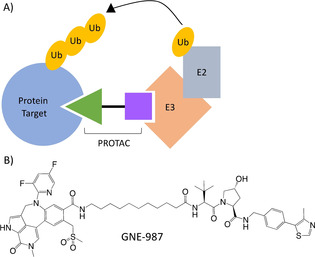
A) PROTACs contain a motif (green) that binds a protein target (blue) separated by a linker from a motif (purple) than binds an E3‐Ubiquitin Ligase (orange). This recruits the E3‐Ubiquitin ligase to the protein target to facilitate ubiquitinylation and degradation of the target by the ubiquitin‐proteasome system.^[1a]^ B) PROTAC GNE‐987, utilised in this study, targets Brd4 bromodomains 1 and 2 for degradation by the E3‐Ubiquitin Ligase Von Hippel Lindau.^[4]^

PROteolysis TArgeting Chimeras (PROTACs) are bifunctional molecules that promote the degradation, rather than inhibition of activity, of a protein target as a therapeutic strategy. These molecules contain a motif (peptide or small molecule) that binds the protein target joined by a chemical linker to a motif that binds an E3‐Ubiquitin ligase (Figure 1A). This allows the recruitment of an E3‐Ubiquitin ligase to the protein target to selectively ubiquitinylate the target and promote its degradation via the cell's endogenous proteasomal degradation machinery.^[1]^ PROTAC activity requires the formation of a ternary complex comprising a target protein, a PROTAC and an E3‐Ubiquitin ligase in a 1 : 1 : 1 subunit stoichiometry.^[2]^ The formation of such ternary complexes can be described by 3‐body binding equilibria.^[3]^ PROTACs have numerous advantageous over canonical drugs that solely inhibit a target. PROTACs can act sub‐stoichiometrically with one PROTAC molecule facilitating the degradation of multiple copies of target protein (as the PROTAC is released following protein degradation) in comparison to the 1 : 1 stoichiometry required for a conventional inhibitor molecule to occupy and inhibit a single protein target molecule. This allows lower dosing regimens, greater therapeutic windows, and reduces the need to maintain high intracellular compound concentrations. PROTACs also require a lower residency time to effectively target proteins for degradation compared to inhibitors required to block activity, allowing weaker, lower affinity sites of the target to be utilized and, thus, the targeting of proteins previously characterized as ‘undruggable’.[^1b^, ^5^] Two PROTACs entered clinical trials in 2019 for the treatment of prostate cancer and breast cancer, with early phase 1 dose escalation studies demonstrating promising initial safety, tolerability and pharmacokinetics for both PROTACs.^[6]^

Despite the advantages of PROTACs, there are many challenges with PROTAC development. An appropriate target with known ligands is required, otherwise an approach for identifying ligands to form part of the PROTAC is a first step.^[7]^ Similarly, following the identification of more of the 600 predicted human E3‐Ubiquitin ligases, screening will be required to discover other ligands that can be utilized as E3‐ubiquitin binding motifs within PROTACs.[^1a^, ^1c^, ^7^, ^8^] To date, the path to producing effective PROTACs is a laborious, time‐consuming, empirical process with high affinity ligands not necessarily leading to potent, if any, target degradation.[^1a^, ^7a^, ^8^, ^9^] A high‐throughput screening approach for detecting stable ternary complexes is currently not available, but is required to develop novel PROTACs.[^7a^, ^9^]

Native MS has routinely been utilized to study protein−protein and protein‐ligand complexes as non‐covalent interactions can be preserved within the mass spectrometer. Various protein‐ligand species and homo‐ and heter‐multimeric protein complexes up to hundreds of kDa in size have been measured by native MS.^[10]^ Native MS provides a quick, label‐free, direct detection method that when combined with automated ESI or nESI sources can allow high‐throughput screening.^[11]^

Here, we utilized native nESI‐MS to study ternary complexes formed by the PROTAC GNE‐987 (Figure 1B) between Brd4 bromodomains 1 and 2 (Brd4^B1^ and Brd4^B2^) and Von Hippel‐Lindau (VHL) E3‐Ubiquitin Ligase. Brd4 is a validated drug target for various cancer implications,^[12]^ whilst VHL has routinely been recruited by PROTACs for the degradation of a diverse range of targets both *in vitro* and *in vivo*.[^5a^, ^13^] GNE‐987 has previously been validated as a more potent Brd4 degraded *in vitro* than standard PROTACs MZ1 and ARV‐771. SPR‐based ternary complex half‐life measurements revealed the Brd4^B1^ ternary complex was more stable than the Brd4^B2^‐containing ternary complex.^[4a]^ GNE‐987 conjugation to an antibody allowed improved *in vivo* stability and pharmacokinetics to allow GNE‐987 to be evaluated for *in vivo* efficacy of Brd4 degradation and tumor regression.^[4]^ A non‐modified high‐resolution SolariX 12 Tesla (12 T) Fourier Transform Ion Cyclotron Resonance Mass Spectrometer (FT‐ICR‐MS) (Bruker) was used to further characterize GNE‐987 ternary complexes. Our high‐resolution native MS allowed us to: 1) directly measure ternary complex formation, 2) provide accurate mass measurements to confirm correct ternary complex stoichiometry, 3) determine all species present at equilibrium (including apo‐subunits and binary PROTAC‐interactions) in one measurement and 4) semi‐quantify the strength and stability of the PROTAC ternary complex based on relative MS intensities.

## Results and Discussion

Brd4 bromodomain 1 (Brd4^B1^), Brd4 bromodomain 2 (Brd4^B2^) and VHL complexed with Elongins B and C (VCB) were visible by native MS with low charge states indicating folded protein (Supporting Figures S1–3). All proteins remained visible with charge states indicative of folded protein upon the addition of 10 % methanol that was utilized for GNE‐987 solubilization (Supporting Figures S1–3). Brd4^B2^, VCB and unbiotinylated Brd4^B1^ had measured MWs consistent with those expected from the protein sequences (Supporting Table S1). The spectra of Brd4^B1^ revealed two protein species present with molecular masses of 18814.5 Da and 19040.5 Da. These two species, with a mass difference of 226.0 Da, correspond to incomplete biotinylation of the protein's avi‐tag (Supporting Table S1).

Binary binding of GNE‐987 to the individual proteins was confirmed via native MS (Supporting Figures S4–6). GNE‐987 bound Brd4^B1^ and Brd4^B2^ with similar affinities. At 5 μM, GNE‐987 bound 40 % Brd4^B1^ and 45 % Brd4^B2^. This increased to 81 % Brd4^B1^ bound and 86 % Brd4^B2^ bound at 20 μM GNE‐987 (Supporting Figures S4 & 5). The similar binding to both Brd4 bromodomains detected by native MS is supported by the previous TR‐FRET binary binding measurements that reported similar IC_50_ values for GNE‐987 of 4.7 nM and 4.4 nM for Brd4^B1^ and Brd4^B2^ respectively.^[4a]^ GNE‐987 was also confirmed to bind VCB, binding 57 % of VCB at 5 μM and 70 % at 20 μM (Supporting Figure S6). The binding response measured by native MS of GNE‐987 to VCB was similar to the binding responses of GNE‐987 to Brd4^B1^ and Brd4^B2^. However, previous cellular nano‐BRET measurements revealed an approximately 130‐fold weaker IC_50_ for VHL compared to the IC_50_ for Brd4^B1^ or Brd4^B2^.^[4a]^ The IC_50_ values are difficult to compare as they were determined via different techniques (cellular nano‐BRET vs recombinant protein TR‐FRET respectively), and the IC_50_ was determined for VHL alone compared to the VCB complex employed here. Despite this, native MS confirmed GNE‐987 was a strong binder of Brd4^B1^, Brd4^B2^ and VCB.

Native MS of equimolar mixtures of Brd4^B1^ or Brd4^B2^ with VCB allowed visualization of both proteins in the one spectrum with the same charge state distributions as when studied individually, in the presence and absence of 10 % methanol (Supporting Figures S7 & 8, Supporting Table S1). Very small amounts of protein−protein interaction between Brd4^B1^ or Brd4^B2^ and VCB without bound PROTAC were detected (approximate ratios between 0.01 and 0.035; data not shown).

**Figure 2:**
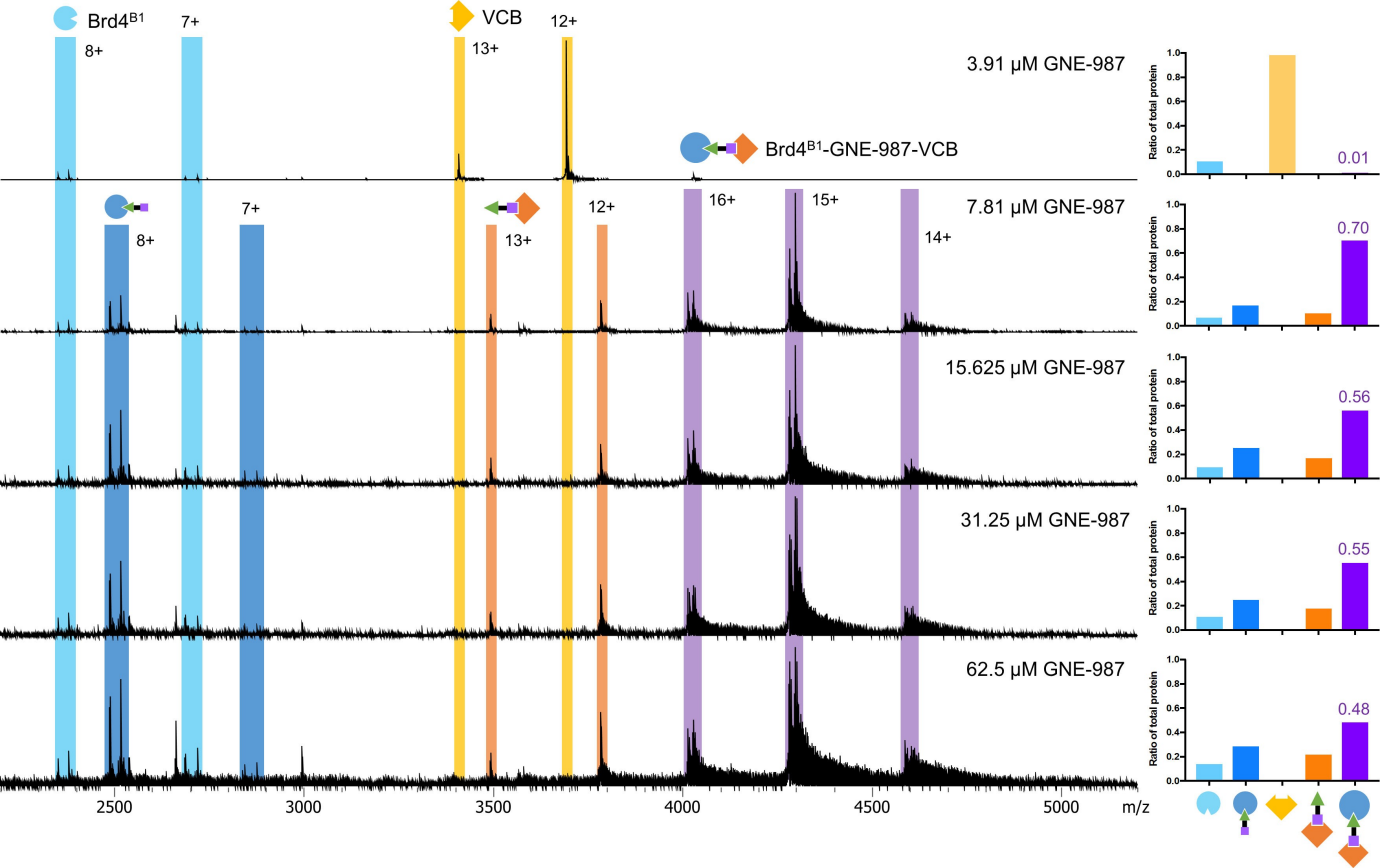
Ternary complex formation between Brd4^B1^, VCB and PROTAC GNE‐987 measured by native nESI‐FT‐ICR‐MS. Apo‐Brd4^B1^ (light blue) and PROTAC‐bound Brd4^B1^ (darker blue) were observed with charge states 7+ and 8+, apo−VCB (yellow) and PROTAC‐bound VCB (orange) were observed with charge states 12+ and 13+, and ternary complex (purple) was detected with charge states 14+, 15+ and 16+. PROTAC concentrations are indicated on the spectra. Quantification of each species as a ratio of total protein in the spectrum is shown on the right.

**Figure 3:**
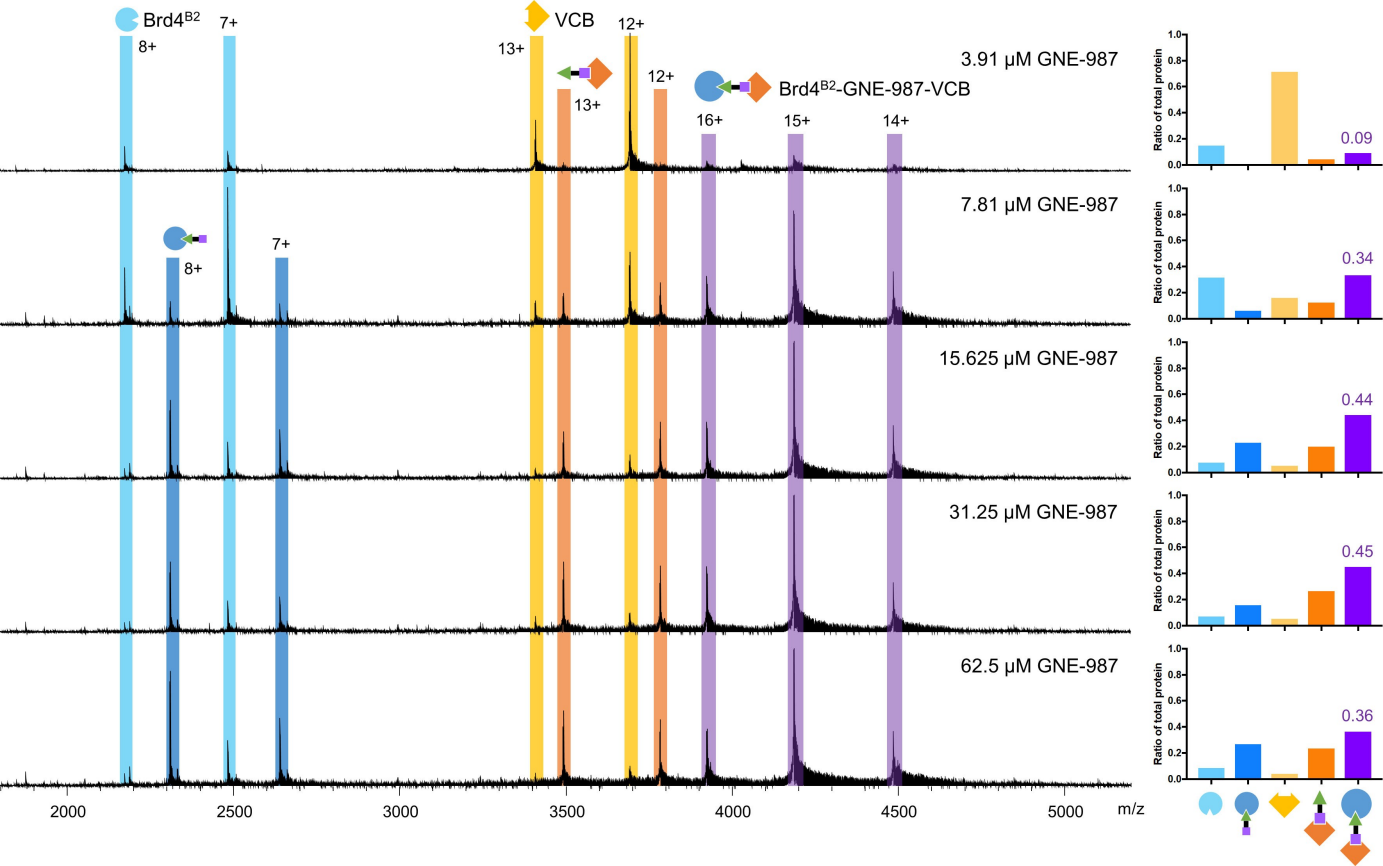
Ternary complex formation between Brd4^B2^, VCB and PROTAC GNE‐987 measured by native nESI‐FT‐ICR‐MS. Apo‐Brd4^B2^ (light blue) and PROTAC‐bound Brd4^B2^ (darker blue) were observed with charge states 7+ and 8+, apo−VCB (yellow) and PROTAC‐bound VCB (orange) were observed with charge states 12+ and 13+, and ternary complex (purple) was detected with charge states 14+, 15+ and 16+. PROTAC concentrations are indicated on the spectra. Quantification of each species as a ratio of total protein in the spectrum is shown on the right.

Ternary complex formation upon the addition of GNE‐987 (at varying concentrations) to an equimolar mixture of Brd4B1 or Brd4B2 and VCB in 10 % methanol was detected by the formation of peaks with m/z 3900–4500 and a charge state distribution of 14+ to 16+, where 15+ was the predominate species (Figures 2 & 3). These ions corresponded to the expected molecular weight of a 1 : 1 : 1 ternary complex of Brd4B1 or Brd4B2, VCB and GNE‐987 (Table 1). These ternary complexes were detected with high mass resolution allowing accurate MW determination of the complex. A ratio of ternary complex formed was calculated by dividing the summed relative abundance of all ternary complex charge states by the summed relative abundance of all charge states of all protein species in the spectrum (Supporting Tables S2 & 3). A maximum ratio of 1 indicated that all protein was incorporated into the ternary complex. This provided a simple strategy for the rough quantification of the protein species, including the ternary complex, present at equilibrium, however, it does require the assumption that all species ionize and are transmitted within the MS with the same efficiency, an idea that has been previously addressed.^[14]^ For Brd4^B1^, GNE‐987 promoted formation of a maximum ternary complex fraction of 0.70 at 7.8 μM GNE‐987 (Table 2, Figure 2). In comparison, a lower ternary complex ratio of 0.34 was formed with 7.8 μM GNE‐987 for Brd4^B2^ (Table 2, Figure 3). The maximum ternary complex fraction formed with Brd4^B2^ was 0.45 (Table 2, Figure 3). This required higher GNE‐987 concentrations of 15.6 μM to 31.25 μM. A larger amount of ternary complex was formed, and with less PROTAC, for Brd4^B1^ compared with Brd4^B2^. This agrees with the previously measured SPR ternary complex half‐life measurements, that revealed the Brd4^B1^‐GNE‐987‐VCB ternary complex has a 100‐fold longer half‐life than the equivalent complex with Brd4^B2^ and, hence, is the more stable ternary complex.^[4a]^ The magnitude of difference between the amount of ternary complex formed for Brd4^B1^ compared to Brd4^B2^ as measured by native MS is not as large as the window of difference in SPR half‐life measurements. Native MS provided a steady state equilibrium measurement whilst SPR measured a real‐time kinetic event, thus each measurement is unique with different factors influencing it.

**Table 1:** Measured molecular masses of the protein species, including ternary complexes, detected from 9 μM Brd4^B1^ or Brd4^B2^, 9 μM VCB and 7.8 μM GNE‐987.

Brd4 Bromodomain	Measured MW [Da]	Corresponding Species	Expected MW [Da]
Brd4^B1^	18814.0	Apo‐Brd4^B1^	18814.4
	19040.0	Biotinylated Apo‐Brd4^B1^	
	19911.7	Holo‐Brd4^B1^	19910.7
	20135.6	Biotinylated Holo‐Brd4^B1^	
	45412.9	Holo‐VCB	45412.7
	64229.7	Ternary complex	64227.1
	64455.5	Ternary complex containing biotinylated Brd4B1	
Brd4^B2^	17386.9	Apo‐Brd4^B2^	17386.8
	18482.4	Holo‐Brd4^B2^	18483.1
	44317.2	Apo‐VCB	44316.4
	45413.0	Holo‐VCB	45412.7
	62799.4	Ternary complex	62799.5

**Table 2:** GNE‐987‐drive ternary complex formation measured by native MS compared to alternative techniques in the literature.

		Brd4^B1^	Brd4^B2^
Ternary complex half‐life t_1/2_ (sec)	SPR^[4a]^	3920±159	39±5
Degradation Activity DC_50_ (nM)	Activity Assays^[4a]^	0.03	
Ratio ternary complex at 7.8 μM	Native MS	0.70	0.34
Maximum ratio ternary complex (and GNE‐987 concentration)	Native MS	0.70 (7.8 μM)	0.44–0.45 (15.6 & 32.5 μM)

Native MS allows the visualization of every species present at equilibrium. At the lowest GNE‐987 concentration tested (3.9 μM) there was minimal ternary complex formation, but there was also very low binary binding to either Brd4^B1^, Brd4^B2^ or VCB (Figure 2 & 3, Supporting Table S3). With the GNE‐987 titration against Brd4^B1^ and VCB, 7.8 μM GNE‐987 resulted in GNE‐987 binding approximately half of the free Brd4^B1^ and all of the free VCB (Figure 2, Supporting Table S3). This GNE‐987 concentration also gave the highest amount of ternary complex. The free holo‐VCB and holo‐Brd4^B1^ could either represent the binary species during the transition to ternary complex, some ternary complex dissociation within the MS, or the PROTAC concentration was too high resulting in excess binary interactions that prevents one PROTAC molecule from facilitating a successful ternary interaction (known as the ‘hook effect’).[^3^, ^5a^, ^15^] As GNE‐987 concentration was further increased, the amount of holo‐Brd4^B1^ continued to increase to a ratio of approximately 1/3 to 1/4 free Brd4^B1^ ligand bound whilst free VCB remained saturated. This trend towards binary saturation of free Brd4^B1^ and VCB, together with a decrease in ternary complex ratio supports the hook effect playing a role. In contrast, binary GNE‐987 binding in Brd4^B2^ samples at 7.8 μM GNE‐987 remained low for free Brd4^B2^ and was less than half the free VCB. This coincided with reasonable ternary complex formation (ratio of 0.34) (Figure 3, Supporting Table S3). At and above15.6 μM GNE‐987, the amount of Brd4^B2^ bound by GNE‐987 was similar to the amount of holo‐Brd4^B1^ at higher GNE‐987 concentrations (∼1/3 to 1/4 total free Brd4^B1^). At this PROTAC concentration the amount of holo‐VCB also increased but binary binding did not saturate VCB as for Brd4^B1^ (∼15–20 % of free VCB, or 5 % of total protein, remained apo). At 15.6 μM and 31.25 μM GNE‐987, the ratio of ternary complex formed was at its highest, before decreasing at 62.5 μM. Monitoring all these species suggests the Brd4^B2^‐containing ternary complex also undergoes the ‘hook effect’ where excess binary binding to the individual proteins prevents one molecule binding both target and E3‐ubiquitin ligase. The ability to measure all species present and monitor the balance of ternary complex formation and binary interactions is a powerful tool for understanding the complex interactions induced by PROTACs.

## Conclusions

Ternary complexes promoted by PROTAC GNE‐987 between Brd4^B1^ or Brd4^B2^ and VHL (here complexed with Elongins B and C to form VCB) were detected by high resolution native MS. This allowed direct confirmation of ternary complex formation with the correct stoichiometry, afforded by the ability to measure the accurate, non‐adducted mass by FT‐ICR‐MS. Adducted complexes have been reported for PROTACs MZ1 and AT1.^[14]^ Native MS revealed greater ternary complex formation for Brd4^B1^ over Brd4^B2^‐containing complexes, which supported previous SPR half‐life measurements that reveal the Brd4^B1^‐containing complex to be more stable, supporting the semi‐quantitative nature of native MS for studying PROTAC ternary complexes.^[4a]^ Native MS allows the direct measurement of all species present at equilibrium providing understanding of PROTAC behavior in regards to target and ligase engagement, the amount of ternary complex formed and the balance between binary and ternary interactions that drive the ‘hook effect’.

Native MS is a label‐free, fast, direct measurement technique for PROTAC development and screening. The use of a nESI source significantly reduced sample consumption relative to alternative techniques for PROTAC studies, with each sample tested here requiring less than 10 μM of each protein and less than 100 μM PROTAC in a volume of 10 μL. The automation of the nESI source allows the automated screening of a 384‐well plate overnight, providing a much‐needed high‐throughput screening approach with direct ternary complex detection for PROTAC development as required in the field.[^7a^, ^9^] Native MS is amenable to studying the binary interactions of PROTAC with target substrate or E3‐Ubiquitin ligase prior to ternary complex formation studies. This allows comparisons between binary interactions and ternary complexes under the same method and sample conditions, of which there are currently few used for PROTAC development (namely SPR and ITC). Native MS screening can also facilitate the challenging PROTAC steps of identification of target ligands and/or novel E3‐Ubiquitin ligase ligands that can be leveraged as binding motifs for PROTACs. Work is ongoing to establish quantitative native MS assays for PROTAC ternary complexes. Current high resolution native MS allows accurate molecular weight determination to confirm complex stoichiometry, relative comparisons of PROTAC ternary complex formation, and provides a screening tool.

## Conflict of interest

The authors declare no conflict of interest.

## Supporting information

As a service to our authors and readers, this journal provides supporting information supplied by the authors. Such materials are peer reviewed and may be re‐organized for online delivery, but are not copy‐edited or typeset. Technical support issues arising from supporting information (other than missing files) should be addressed to the authors.

Supplementary
