# Supplementary material for: Native Mass Spectrometry for the Study of PROTAC GNE‐987‐Containing Ternary Complexes
Source: ChemMedChem. 2021 May 4;16(14):2206–10. doi: 10.1002/cmdc.202100113 (PMC8359942; doi:10.1002/cmdc.202100113)
Supplement: Supplementary file 1 — Supplementary [file CMDC-16-2206-s001.pdf]

ChemMedChem

Supporting Information

Native Mass Spectrometry for the Study of PROTAC GNE-987-Containing Ternary Complexes

Louise M. Sternicki, Jim Nonomiya, Miaomiao Liu, Melinda M. Mulvihill,* and Ronald J. Quinn*

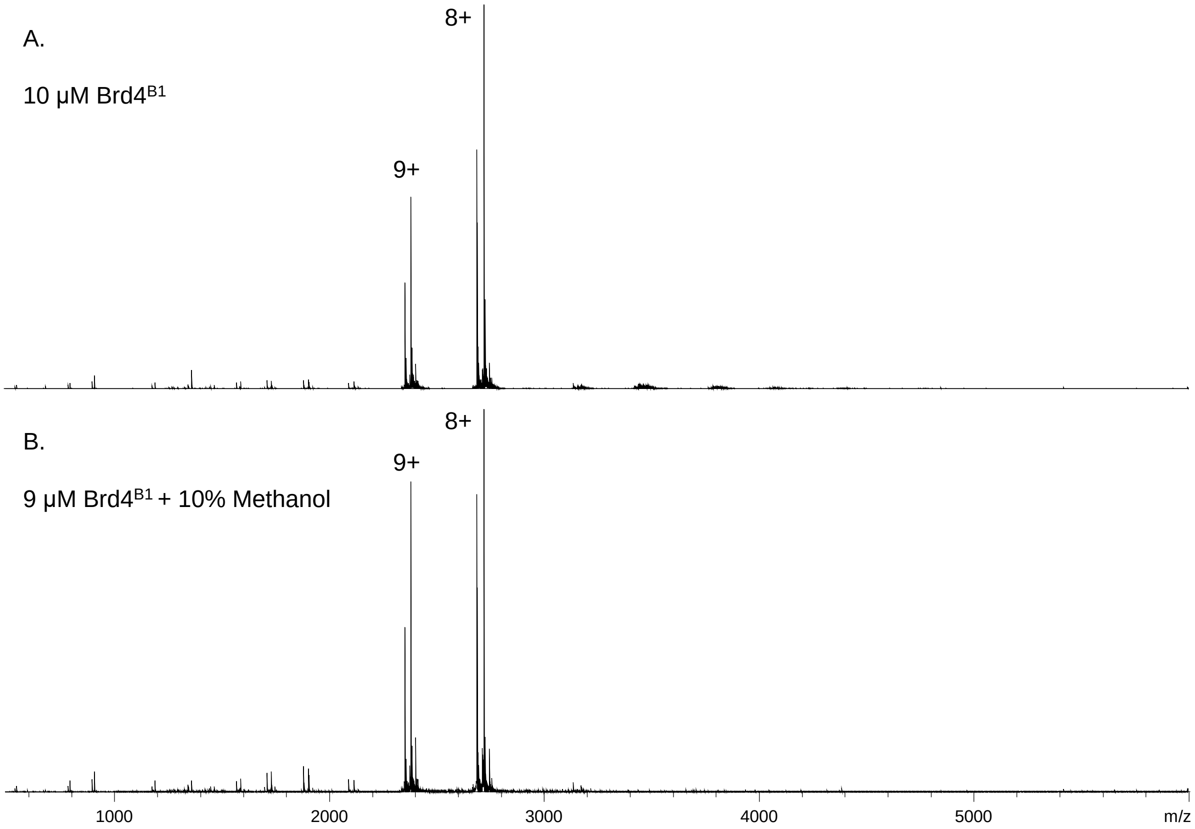

Figure S1. Brd4^{B1} (9 µM) is visible in its native, folded state by high resolution native FT-ICR-MS both A) without solvent and in the presence of B) 10% methanol.

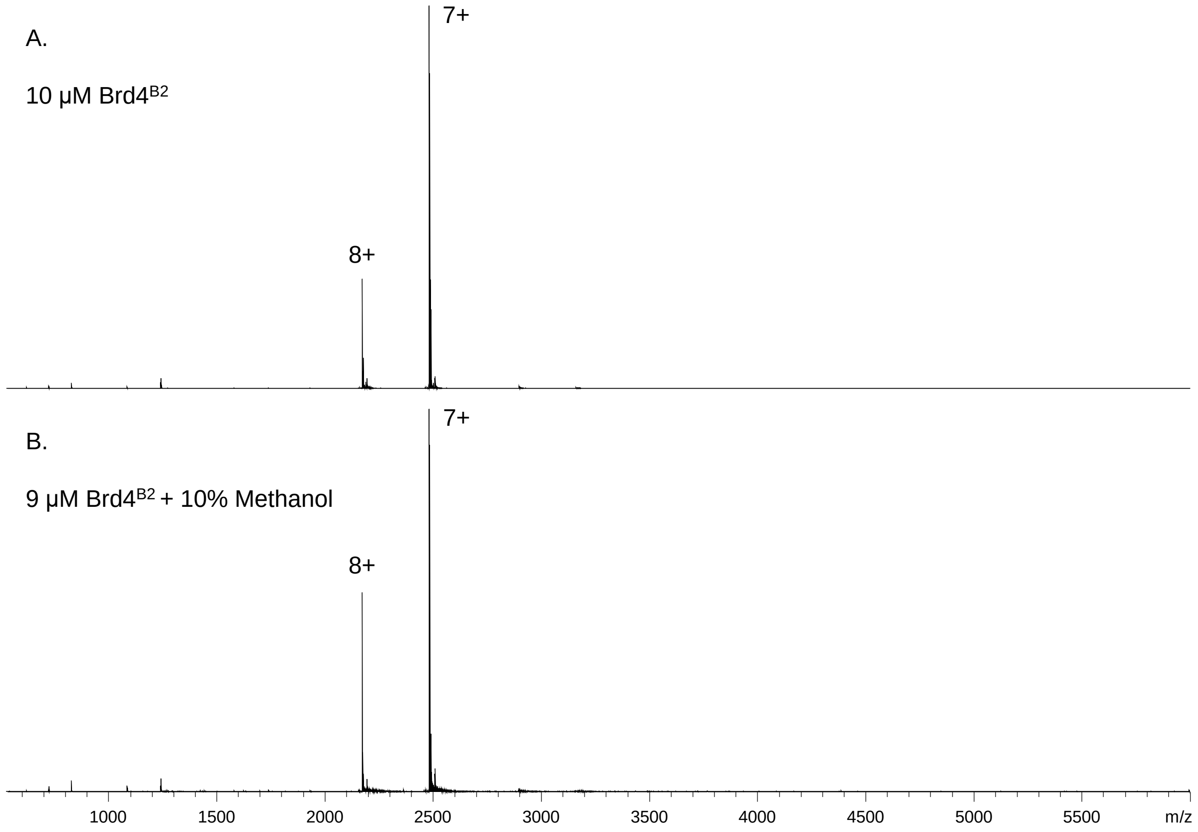

Figure S2. Brd4^{B2} (9 µM) is visible in its native, folded state by high resolution native FT-ICR-MS both A) without solvent and in the presence of B) 10% methanol.

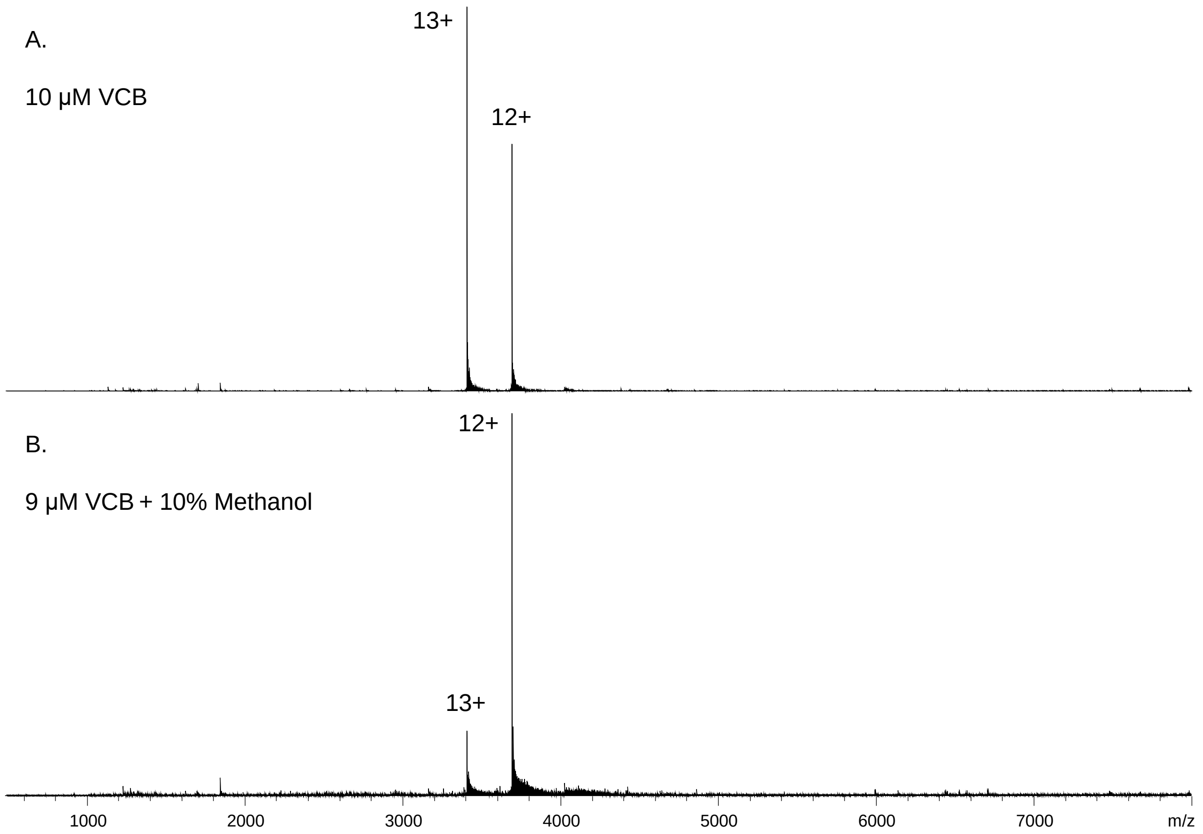

Figure S3. E3-ubiquitin ligase VHL (co-expressed with elongins B and C) (termed VCB) (9 µM) is visible in its native, folded state by high resolution native FT-ICR-MS both A) without solvent and in the presence of B) 10% methanol.

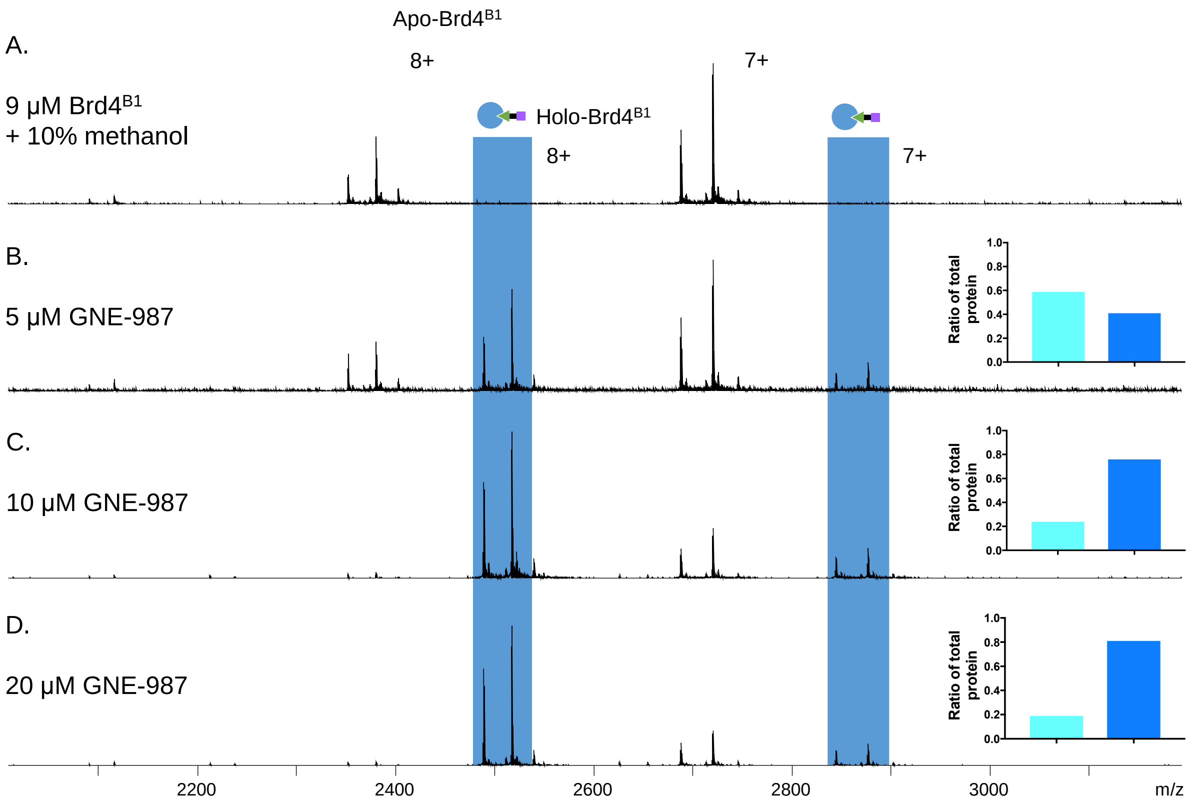

Figure S4. Binary binding of GNE-987 to 9 µM Brd4^{B1}. Light blue highlighting denotes apo-Brd4^{B1} whilst the darker blue highlighting indicates PROTAC-bound Brd4^{B1}. Quantitation of apo-Brd4^{B1} and holo-Brd4^{B1} as a ratio of total protein are shown to the right of the spectra. Data represent a single experiment.

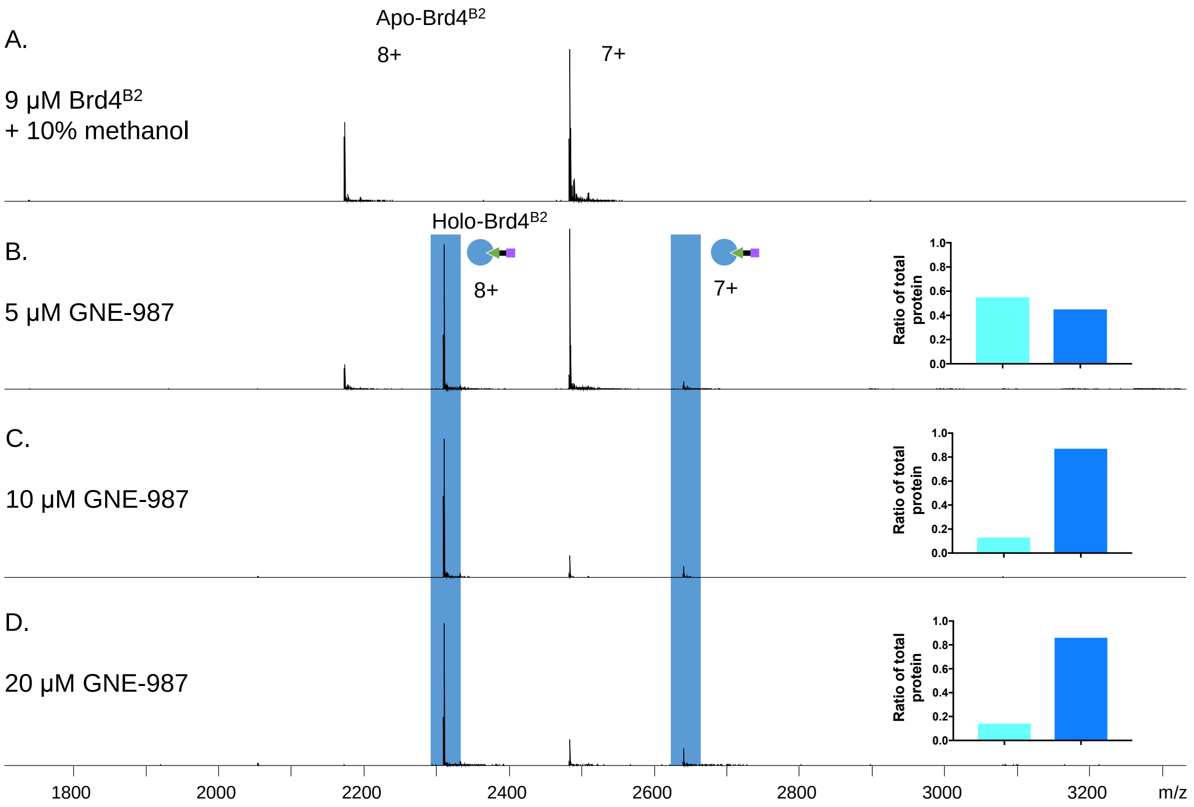

Figure S5. Binary binding of GNE-987 to 9 μM Brd4^{B2}. Light blue highlighting denotes apo-Brd4^{B2} whilst the darker blue highlighting indicates PROTAC-bound Brd4^{B2}. Quantitation of apo-Brd4^{B2} and holo-Brd4^{B2} as a ratio of total protein are shown to the right of the spectra. Data represent a single experiment.

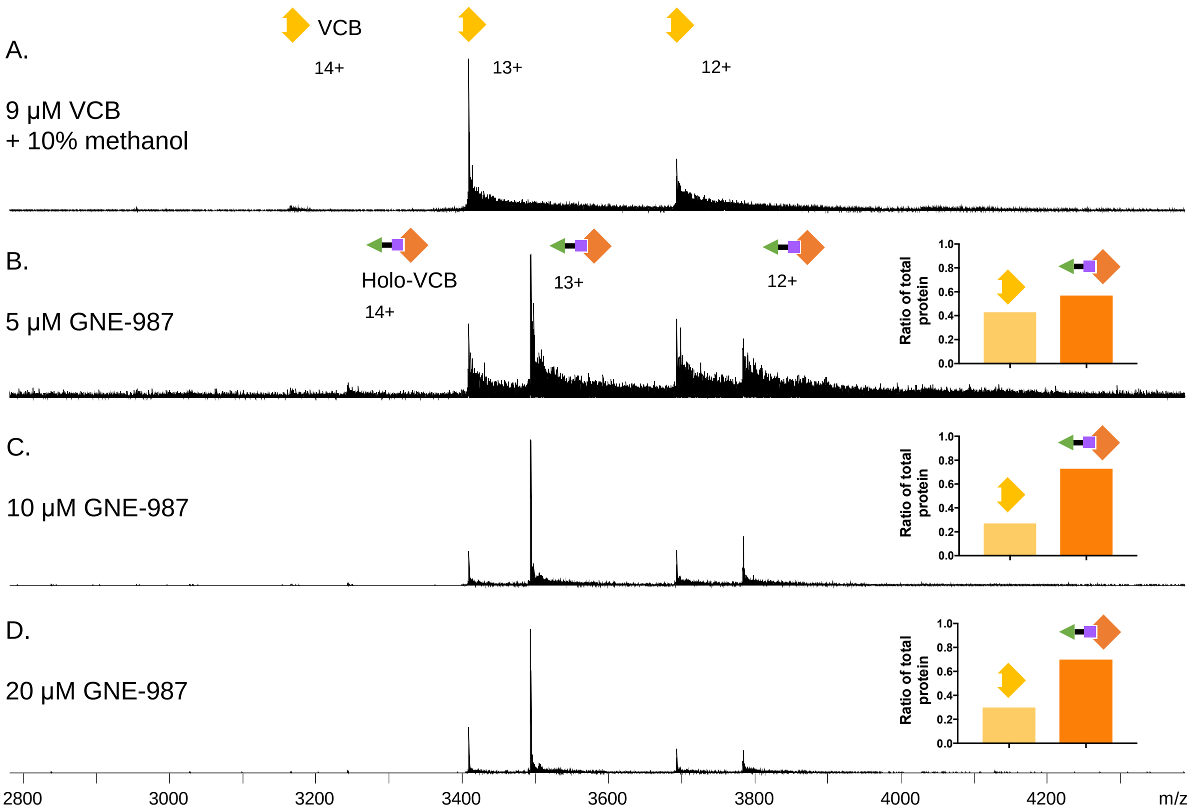

Figure S6. Binary binding of GNE-987 to 9 μM VCB. Yellow highlighting denotes apo-VCB whilst orange highlighting indicated PROTAC-bound VCB. Quantitation of apo-VCB and holo-VCB as a ratio of total protein are shown to the right of the spectra. Data represent a single experiment.

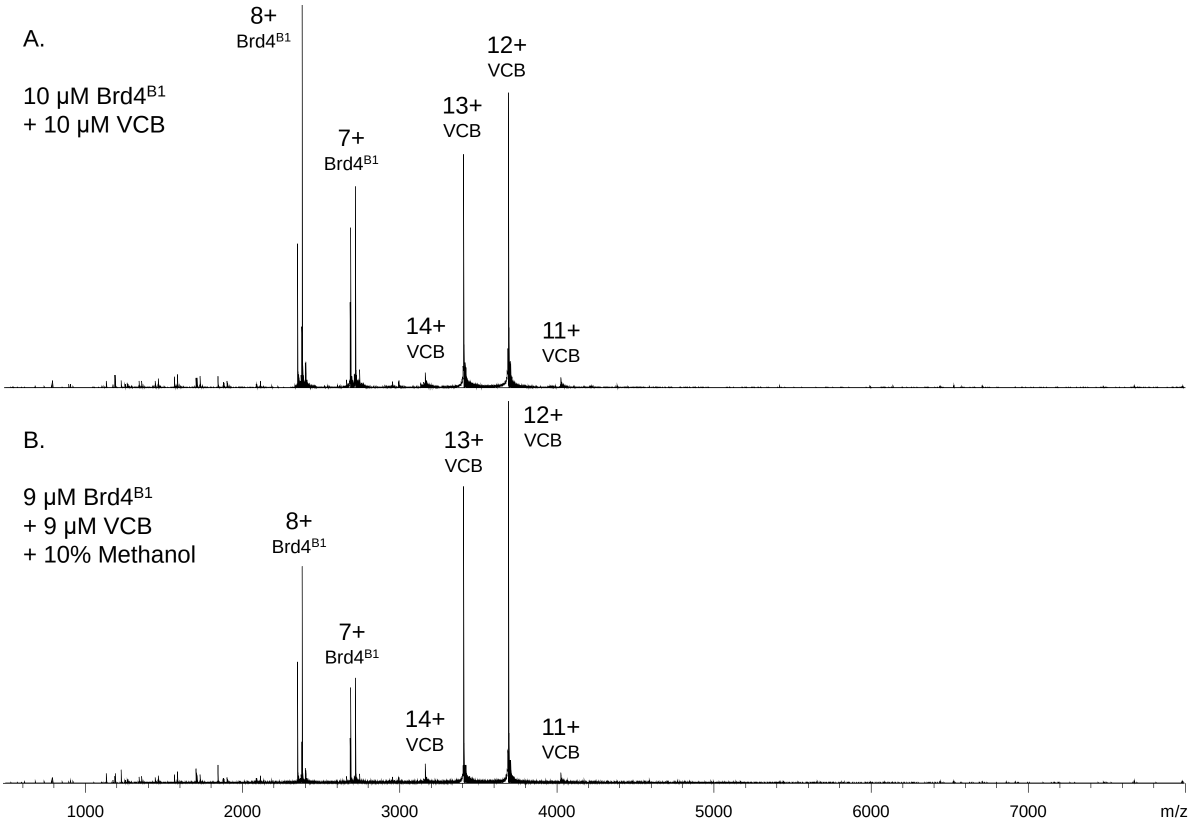

Figure S7. Reference spectrum of an equimolar mixture of Brd4[B1] and VCB both A) without solvent and in the presence of B) 10% methanol.

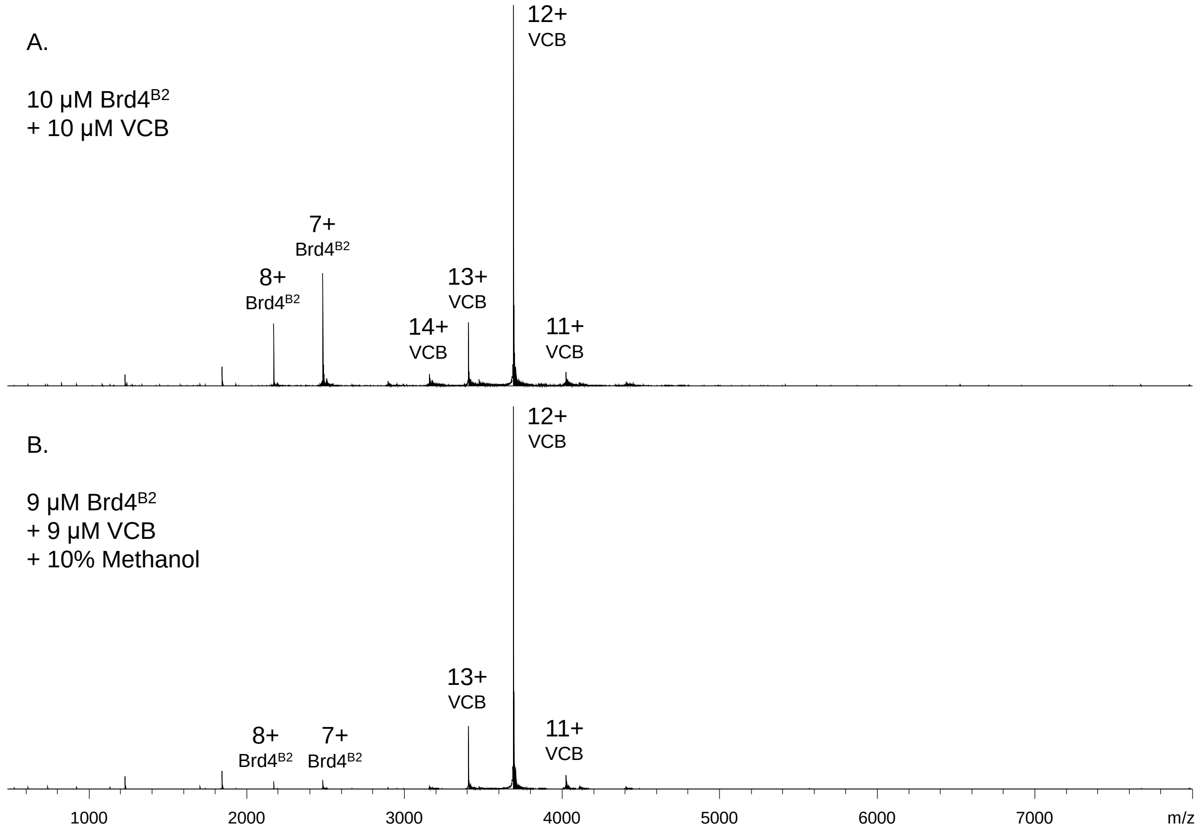

Figure S8. Reference spectrum of an equimolar mixture of Brd4^{B2} and VCB both A) without solvent and in the presence of B) 10% methanol.

Table S1. Measured molecular masses of Brd4^{B1}, Brd4^{B2} and VCB.

Protein	Figure	Experimental Conditions	Measured MW (Da)	Corresponding Species	Expected MW (Da)
Brd4^{B1}	S1A	9 μM, no solvent	18815.7	Brd4^{B1}	18814.4
			19040.8	Biotinylated Brd4^{B1}	
	S1B	9 μM, 10% methanol	18813.8	Brd4^{B1}	18814.4
			19039.9	Biotinylated Brd4^{B1}	
Brd4^{B2}	S2A	9 μM, no solvent	17387.5	Brd4^{B2}	17386.8
	S2B	9 μM, 10% methanol	17386.6	Brd4^{B2}	17386.8
VCB	S3A	9 μM, no solvent	44318.2	VCB	44316.4
	S3B	9 μM, 10% methanol	44317.6	VCB	44316.4
Brd4^{B1} + VCB	S7A	9 μM Brd4^{B1}, 9 μM VCB, no solvent	18815.1	Brd4^{B1}	18814.4
			19040.0	Biotinylated Brd4^{B1}	
			44317.0	VCB	44316.4
	S7B	9 μM Brd4^{B1}, 9 μM VCB, 10% methanol	18815.1	Brd4^{B1}	18814.4
			19039.9	Biotinylated Brd4^{B1}	
			44318.8	VCB	44316.4
Brd4^{B2} + VCB	S8A	9 μM Brd4^{B2}, 9 μM VCB, no solvent	17387.9	Brd4^{B2}	17386.8
			44316.2	VCB	44316.4
	S8B	9 μM Brd4^{B2}, 9 μM VCB, 10% methanol	17384.8	Brd4^{B2}	17386.8
			44318.9	VCB	44316.4

Table S2. Fraction ternary complex formed between Brd4^{B1} or Brd4^{B2}, VCB and GNE-987 across all PROTAC concentrations tested. Values for Brd4^{B1} are the average of two independent replicates +/- SEM, whilst values for Brd4^{B2} are from a single determinate.

**Brd4 Bromodomain**	**Fraction Ternary Complex at all GNE-987 concentrations**				
	**3.91 μM**	**7.81 μM**	**15.625 μM**	**31.25 μM**	**62.5 μM**
Brd4^{B1}	0.01	0.70	0.56	0.55	0.48
Brd4^{B2}	0.09	0.34	0.44	0.45	0.36

Table S3. Ratios of all protein species for mixtures of Brd4^{B1} or Brd4^{B2}, VCB and GNE-987 across all PROTAC concentrations tested. Mixtures with Brd4^{B1} are the average +/- SEM of two independent replicates, whilst mixtures with Brd4^{B2} represent one independent experiment. Note the sum of all ratios in a single spectrum may not equal 1 as other protein species were detected (i.e. multiple PROTAC binding events, particularly binary binding to VCB, and protein-protein interactions between Brd4$^{B1 \text{ or } B2}$ and VCB in the absence of PROTAC).

Brd4 Bromodomain	Protein Species	Species Ratios at all GNE-987 concentrations				
		3.91 μM	7.81 μM	15.625 μM	31.25 μM	62.5 μM
Brd4^{B1}	Apo-Brd4^{B1}	0.11	0.07	0.09	0.11	0.14
	Holo-Brd4^{B1}	0	0.15	0.23	0.22	0.24
	Apo-VCB	0.88	0	0	0	0
	Holo-VCB	0	0.08	0.11	0.12	0.13
	Ternary Complex	0.01	0.70	0.56	0.55	0.48
Brd4^{B2}	Apo-Brd4^{B2}	0.15	0.32	0.08	0.07	0.09
	Holo-Brd4^{B2}	0	0.06	0.23	0.16	0.27
	Apo-VCB	0.71	0.16	0.05	0.05	0.04
	Holo-VCB	0.05	0.12	0.20	0.26	0.24
	Ternary Complex	0.09	0.34	0.44	0.45	0.36

Materials and Methods

PROTAC synthetic chemistry
The PROTAC GNE-987 was synthesized as previously reported. [1]

Recombinant production of proteins Brd4^{B1}, Brd4B2, and VCB
Production of proteins Brd4^{B1}, Brd4^{B2} and VCB (VHL complexed with Elongins B and C) were completed as previously outlined. [1a]

Native MS to measure PROTAC GNE-987 binary binding and ternary complex formation
Protein was buffer exchanged into 150 mM ammonium acetate pH 6.9-7.0 via Illustra NAP-5 Columns (GE Healthcare Life Sciences) to a final concentration of 20 µM. GNE-987 stock solubilised in DMSO was plated into 384-well microtiter plates at 10-fold higher concentrations than required. DMSO was removed from the microtiter plates via freeze-drying. PROTACs were resolubilized in methanol before being diluted 1/10 by the addition of buffer exchange protein in 150 mM ammonium acetate pH 6.9-7.0. Final samples for native MS contained 9 µM protein/s and 10% methanol, with the addition GNE-987 to the specified concentration (i.e. 5 to 20 µM for binary interactions, 3.9 µM to 62.5 µM for ternary complexes). Initially, ternary complex formation was measured with PROTAC titrated across a broad concentration range from 0.488 µM to 1000 µM to allow determination of the optimal concentrations for ternary complex formation (i.e. 3.9 µM to 62.5 µM).
Proteins were introduced into a SolariX XR 12 T FT-ICR-MS (Bruker) via nESI using a TriVersa NanoMate (Advion) as the source. nESI chips containing nozzle emitters with a 5.5 µm internal diameter (Advion) were utilised for sample introduction. MS data were acquired in the positive ion mode from 200 *m/z* to either 6000, 8000 or 10,000 *m/z*. Parameters were tuned to achieve the highest signal intensity whilst maintaining protein-ligand complexes. NanoMate source parameters were; voltage: 1.7 kV, gas pressure: 1 psi. FT-ICR-MS parameters were as follows; capillary: 1000 V, end plate offset: 0 V, dry gas: 1.5 L/min, dry temperature: 75-100 °C; nebulizer: 2 bar, capillary exit: 220 V, deflector plate: 250 V, skimmer 1: 30 V, collision voltage: -5 to -15 V, time of flight: 1.75 ms, size: 4 M, scans: 16, ion acquisition time: 4 seconds.
Data was acquired using Compass Control Software (Bruker) and analyzed with Compass Data Analysis (Bruker), Microsoft Excel and GraphPad Prism. The fraction (or ratio) of a specific protein species was calculated as the total of the peak intensities of all charge states of that species divided by the total of peak intensities of all charge states of all proteins present in the spectrum.

References

[1] aT. H. Pillow, P. Adhikari, R. A. Blake, J. Chen, G. Del Rosario, D. Gauri, I. Figueroa, K. E. Gascoigne, A. V. Kamath, S. Kaufman, T. Kleinheinz, K. R. Kozak, B. Latifi, D. D. Leipold, C. Sing Li, R. Li, M. M. Mulvihill, A. O'Donohue, R. K. Rowntree, J. D. Sadowsky, J. Wai, X. Wang, C. Wu, Z. Xu, H. Yao, S.-F. Yu, D. Zhang, R. Zhang, H. Zhang, H. Zhou, X. Zhu, P. Dragovich, *ChemMedChem* **2020**, *15*, 17-25; bP. S. Dragovich, T. H. Pillow, R. A. Blake, J. D. Sadowsky, E. Adaligil, P. Adhikari, J. Chen, N. Corr, J. dela Cruz-Chuh, G. Del Rosario, A. Fullerton, S. J. Hartman, F. Jiang, S. Kaufman, T. Kleinheinz, K. R. Kozak, L. Liu, Y. Lu, M. M. Mulvihill, J. M. Murray, A. O'Donohue, R. K. Rowntree, W. S. Sawyer, L. R. Staben, J. Wai, J. Wang, B. Wei, W. Wei, Z. Xu, H. Yao, S.-F. Yu, D. Zhang, H. Zhang, S. Zhang, Y. Zhao, H. Zhou, X. Zhu, *J. Med. Chem.* **2021**, *64*, 2576-2607.
